# Fan beam computed tomography-guided online adaptive external radiotherapy for cervical cancer achieves pathological complete response: A case report

**DOI:** 10.3892/ol.2025.15090

**Published:** 2025-05-14

**Authors:** Haibo Peng, Ningyue Xu, Dong Gao, Huigang Tan, Tao Ren

**Affiliations:** 1 Department of Oncology, School of Clinical Medicine and The First Affiliated Hospital of Chengdu Medical College, Chengdu, Sichuan 610500, P.R. China; 2 Clinical Key Speciality (Oncology Department) of Sichuan Province, Chengdu, Sichuan 610500, P.R. China; 3 United Imaging Central Research Institute Co., Ltd., Shanghai 201807, P.R. China; 4 Radiology and Therapy Clinical Medical Research Center of Sichuan Province, Chengdu, Sichuan 610500, P.R. China; 5 Department of Oncology, The First Affiliated Hospital of Traditional Chinese Medicine of Chengdu Medical College, Xindu Hospital of Traditional Chinese Medicine, Chengdu, Sichuan 610500, P.R. China

**Keywords:** cervical cancer, online adaptive radiotherapy, image-guided radiotherapy, intelligent segmentation, *in vivo* dose validation

## Abstract

Radiotherapy (RT) is an established viable treatment for cervical cancer across all clinical stages. However, the therapeutic ratio of conventional techniques remains suboptimal due to the anatomical proximity of the cervix to critical pelvic organs. The current study presents a case of pathological complete response (pCR) achieved exclusively through preoperative online adaptive RT (oART) guided by fan beam computed tomography (FBCT). A 54-year-old woman presenting with irregular vaginal bleeding was diagnosed with a cervical mass via pelvic imaging. Subsequent histopathological biopsy confirmed invasive papillary squamous cell carcinoma. Preoperative evaluation, supplemented by laboratory and imaging studies, ruled out distant metastases. The patient underwent fractionated oART using a CT-linear accelerator platform. Post-treatment imaging demonstrated complete resolution of the lesion, and surgical histopathology revealed no residual malignancy. This case highlights the feasibility, safety and dosimetric precision of FBCT-guided oART in cervical cancer. The pCR achieved in this case indicates that oART has the potential to improve the treatment of cervical cancer.

## Introduction

Recent epidemiological data confirm increasing global incidence (13.83 cases per 100,000 individuals) and mortality (4.54 deaths per 100,000 individuals) rates of cervical cancer, as evidenced by updated cancer surveillance reports (1). External beam radiotherapy (EBRT), often combined with brachytherapy (BT), remains a cornerstone treatment. Standard EBRT protocols involve ~25 fractions delivered over 4–6 weeks. During this extended treatment course, anatomical shifts in surrounding organs (e.g., bladder and rectum) and uterine mobility, coupled with tumor deformation and volumetric regression, are frequently observed. To mitigate these dynamic changes and setup uncertainties, conventional RT planning expands the clinical target volume (CTV) by 7- to 15-mm margins to create planning target volumes (PTVs), with dose prescriptions based on PTV contours. However, studies indicate that CTV displacement in cervical cancer may exceed 20 mm during treatment (2), suggesting that conventional PTV margins may inadequately account for such variability. Furthermore, clinics often apply uniform PTV margins across patient cohorts, despite significant inter-individual differences in organ-at-risk (OAR) displacement, uterine motility, tumor regression rates and daily setup errors. Suboptimal margins risk geographic miss (underdosing targets) or excessive irradiation of healthy tissues, contributing to treatment-related toxicities such as gastrointestinal complications, which affect ~70% of patients with EBRT (3). While image-guided RT (IGRT) reduces setup inaccuracies, it cannot compensate for interfractional organ and tumor variations. Moreover, standard three-degree-of-freedom treatment couches lack rotational correction capabilities, further limiting positional accuracy.

Online adaptive RT (oART) was first conceptualized by Yan *et al* (4) in 1997 to address discrepancies between preplanned treatments and real-time anatomical changes in targets and OARs during RT (5). The core principle of oART lies in its dynamic closed-loop framework, which integrates daily imaging, self-responding adjustments and self-correcting adaptations. Recent advances in imaging, computational power and artificial intelligence (AI) have enabled its clinical implementation. A typical oART workflow comprises daily image acquisition, deformable image registration, automated segmentation of targets and OARs, adaptive plan generation, dosimetric evaluation, *in vivo* quality assurance (QA) and beam delivery (6). This process demands robust AI algorithms, multidisciplinary team coordination and high-performance RT systems. Owing to rapid technological innovations, oART has emerged as a pivotal research focus in contemporary RT, offering the potential to enhance precision and adaptability in cancer treatment.

Clinical evidence positions cervical cancer as an optimal candidate for oART. In the present study, oART was applied to all fractions during the EBRT phase for a patient with cervical cancer. All aspects of the oART process were observed and studied. Dosimetric parameters and imaging before and after RT were compared. More importantly, a result of pathological complete response (pCR) was achieved in this case only after implementing external beam oART.

## Case report

**Figure 1.**
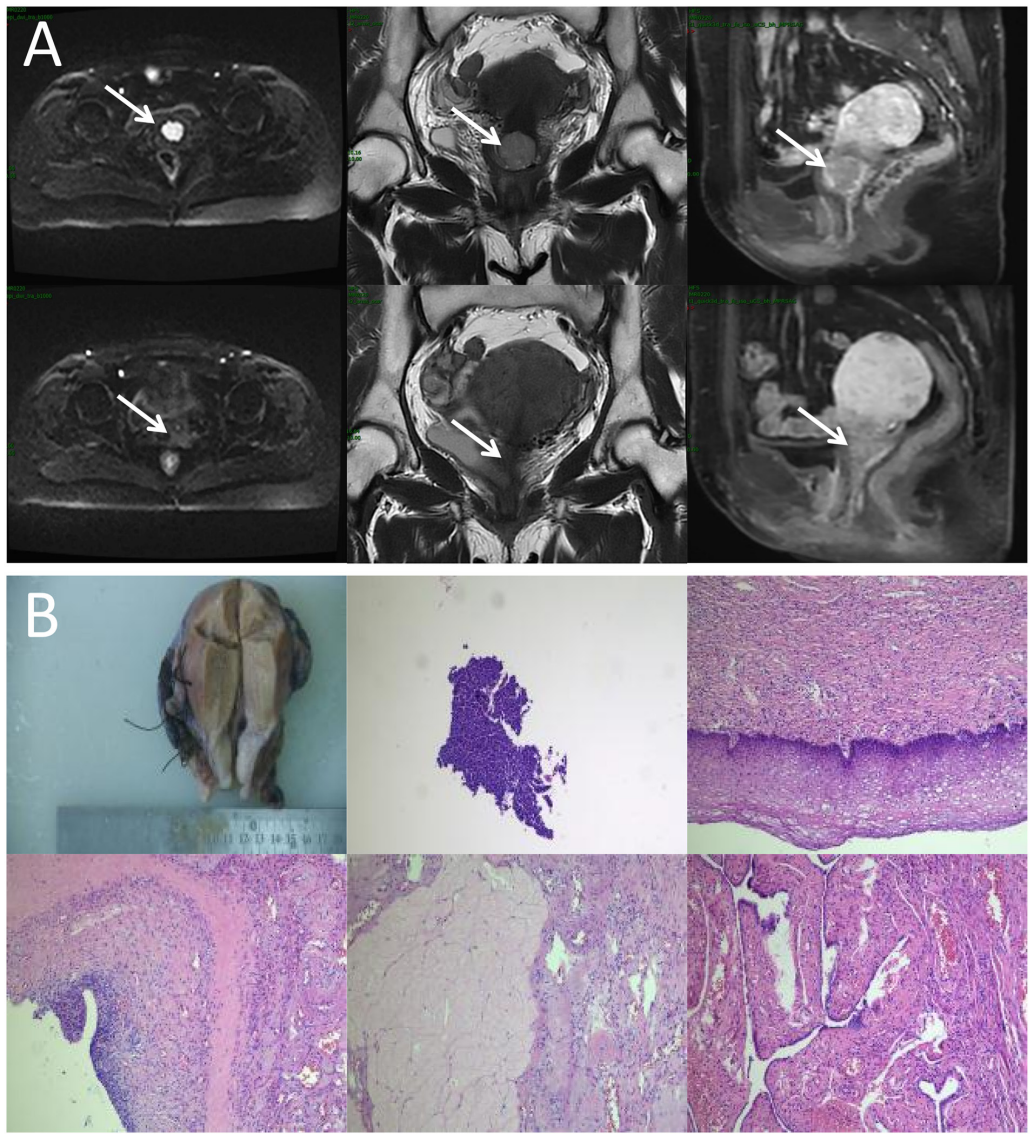
Locoregional response evaluation. (A) Magnetic resonance imaging of the patient pre-radiotherapy (upper) and post-radiotherapy (lower). From left to right: Diffusion-weighted imaging (b=1,000 sec/mm^2^) axial view, T2-weighted coronal plane and T1-weighted sagittal plane. Arrows indicate the mass. (B) Representative imaging from the postoperative histopathological diagnosis report, comprising both the gross surgical specimen and photomicrographs (obtained from the cervix, surgical margins, ovaries, fallopian tubes and pelvic lymph nodes; hematoxylin and eosin staining, with magnifications of ×40, ×100, ×200, ×200 and ×400, respectively).

**Figure 2.**
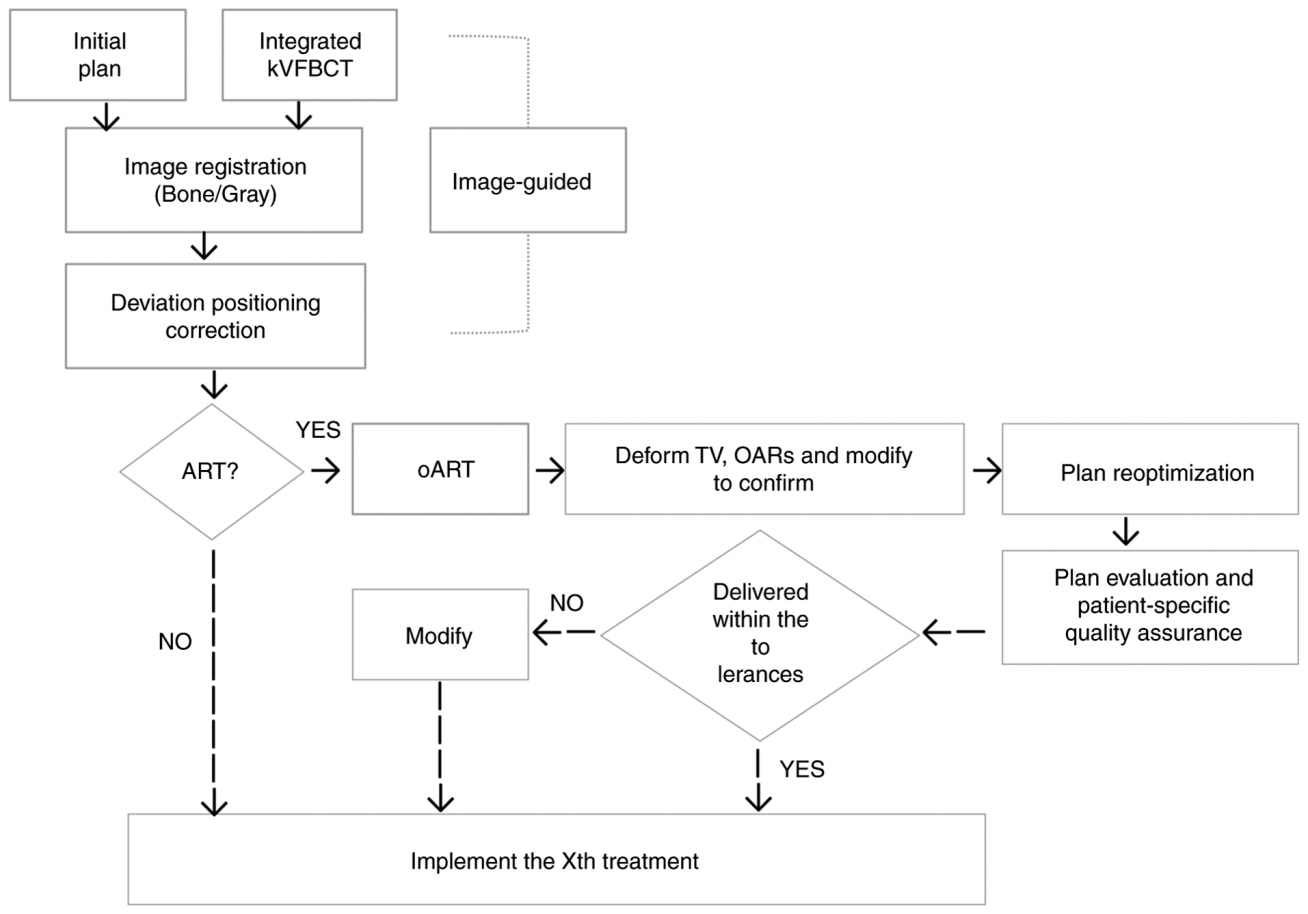
Flow chart of FBCT-guided oART. oART, online adaptive radiotherapy; kV, kilovoltage; FBCT, fan beam computed tomography; OAR, organ at risk; TV, target volume.

A previously healthy 54-year-old woman experienced irregular vaginal bleeding for 1 month and initially presented at The First Affiliated Hospital of Chengdu Medical College (Chengdu, China) in June 2023. Magnetic resonance imaging (MRI) showed that there was a clump of slight hypointensity on T1WI and slight hyperintensity on T2WI in the cervical cavity, with a size of ~2.2×2.4×1.9 cm. The mass involved the lower one-third of the uterine body and protruded to the lower part of the uterine cavity, and the lower edge was not clearly demarcated with the posterior wall of the upper end of the vagina (Fig. 1A). After gynecological examination and biopsy, the pathological diagnosis of papillary squamous cell carcinoma was confirmed by the Department of Pathology. Combined with laboratory examinations (including complete blood count, hepatic and renal function tests) and imaging examinations [including computed tomography (CT) and MRI.], the general condition of the patient was comprehensively evaluated, and with the consent of the patient, the treatment regimen of external RT combined with brachyradiotherapy was adopted, with concurrent chemotherapy (380 mg albumin-bound paclitaxel on day 1 + 110 mg nedaplatin on day 1; every 3 weeks). According to the Radiation Therapy Oncology Group guidelines (7,8), the primary gross tumor volume (GTVp), CTV (included the tumor, cervix, uterine corpus, parametrium, upper vaginal portion, and lymph node regions, including the common iliac, internal iliac, external iliac, obturator and presacral areas) and PTV (created by expanding CTV with 5-mm margins) were contoured. Subsequently, 95% of the PTV received 100% of the prescription dose (50.4 Gy/28 fractions/6 weeks). oART based on fan-beam CT (FBCT) guidance was conducted for every fraction of EBRT for this patient. A flow chart of the oART is illustrated in Fig. 2.

After completing EBRT, the image review of MRI was performed and the results showed that the lesions in the cervix region had basically regressed (Fig. 1A). No significant radiotherapy-related toxicity or side effects occurred throughout the process for the patient. At 1-month post-EBRT, the patient asked for a change in treatment and opted for surgery instead of BT. There were no significant intestinal adhesions and parametrial tissue thickening during the operation. The surgery was successfully completed, no cancer cells were observed in the postoperative pathological examination, and a result of pCR was achieved. In the follow-up of nearly 1 year (final assessment in October 2024), the patient did not complain of obvious discomfort, and there was no obvious abnormality in the results of various examinations.

## Discussion

**Figure 3.**
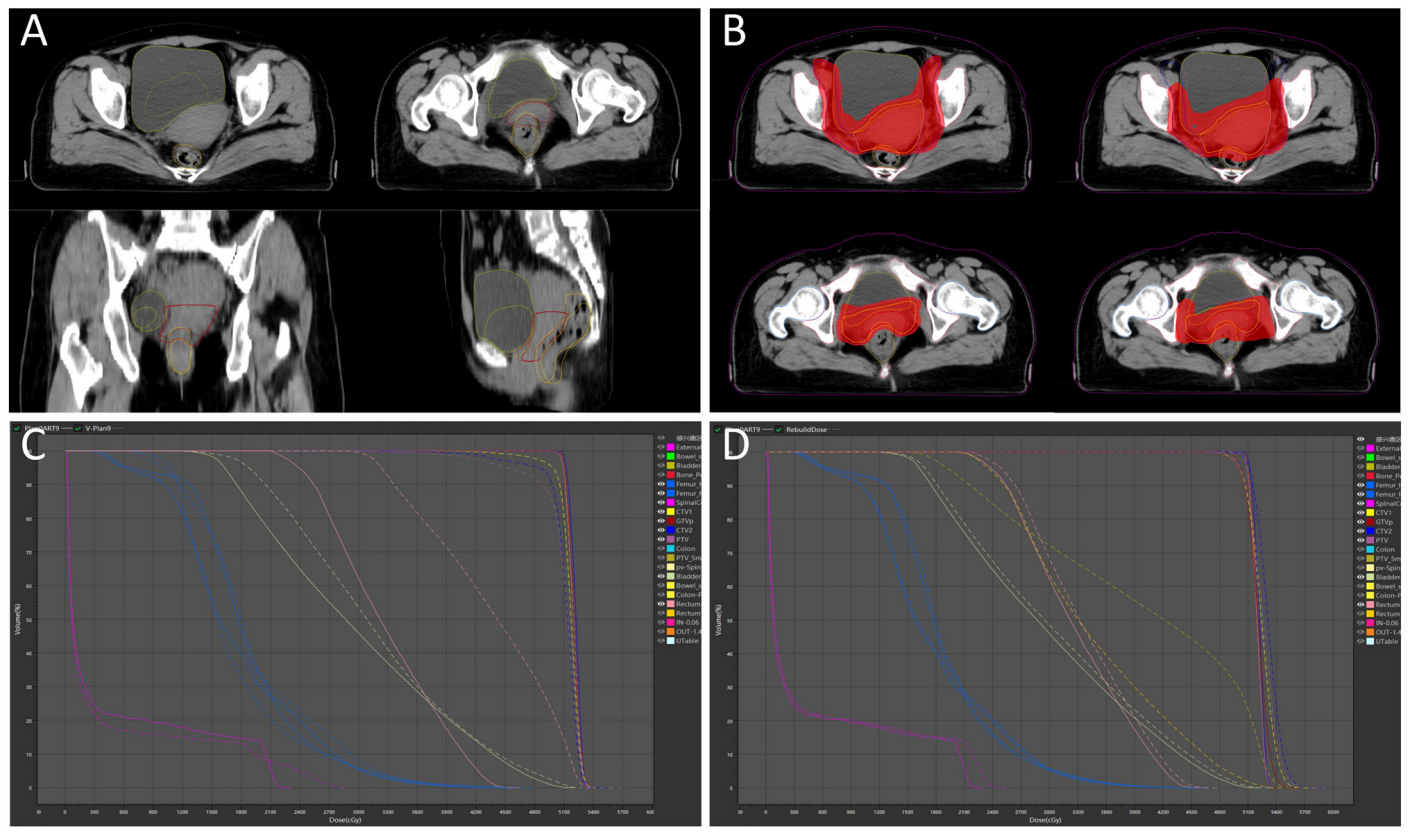
Effect of rectal, bladder and lesion volume changes on dose distribution. (A) The changes of volume in bladder, rectum and GTVp between the images of 9th fraction and positioning computed tomography. (B) Difference in dose distribution between the 9th fractional ART-Plan and IGRT-Plan. (C) The DVH comparison of the 9th fractional ART-Plan and IGRT-Plan. (D) The DVH comparison between 9th fractional ART-Plan and *in vivo* dose verification of 3D reconstruction. GTVp, primary gross tumor volume; DVH, dose-volume histogram; IGRT, image-guided radiotherapy; ART, adaptive radiotherapy.

RT is a cornerstone of cervical cancer treatment. However, the proximity of the cervix to the bladder and rectum introduces challenges: During successive treatment fractions, variations in the morphology and position of these organs occur to varying degrees, leading to discrepancies between the delivered dose and the planned dose. In the present case, the bladder and rectum exhibited significant and irregular morphological and volumetric changes. Concurrently, the volume of GTVp progressively decreased (Fig. 3A), directly altering the actual dose distribution of the initial plan. oART addressed these issues by reoptimizing the dose distribution to conform to the target anatomy. This approach not only mitigated the impact of inter-fractional anatomical shifts and rotational deviations but also allowed for a reduced CTV to PTV expansion margin. Consequently, both target coverage and OARs dose constraints were optimized (Fig. 3B). Compared with the IGRT plan (Fig. 3C), the ART plan demonstrated superior dosimetric outcomes for target coverage and OAR sparing (9,10) (Table I). For instance, anatomical deformations were observed in the bladder, rectum and GTVp during the ninth treatment fraction in the present case. Marked clinical concerns arose when applying IGRT based on the original plan, including an increased irradiated rectal volume and a partial target miss in the ventral tumor region. After adaptive plan optimization, the volume ratio of 40 Gy covering the rectum (V_40Gy_) was significantly reduced from 67.57 to 13.21%, and the PTV coverage (V_100%_) increased from 77.63 to 99.00%. The PTV coverage (V_100%_) values for the 28 ART plans and IGRT plans were 98.51±0.82 and 88.99±4.40%, respectively. Although this case study did not include statistical analyses to account for random variability, the ART plans demonstrated clear dosimetric superiority, evidenced by exceptional consistency and clinical target achievement.

**Figure 4.**
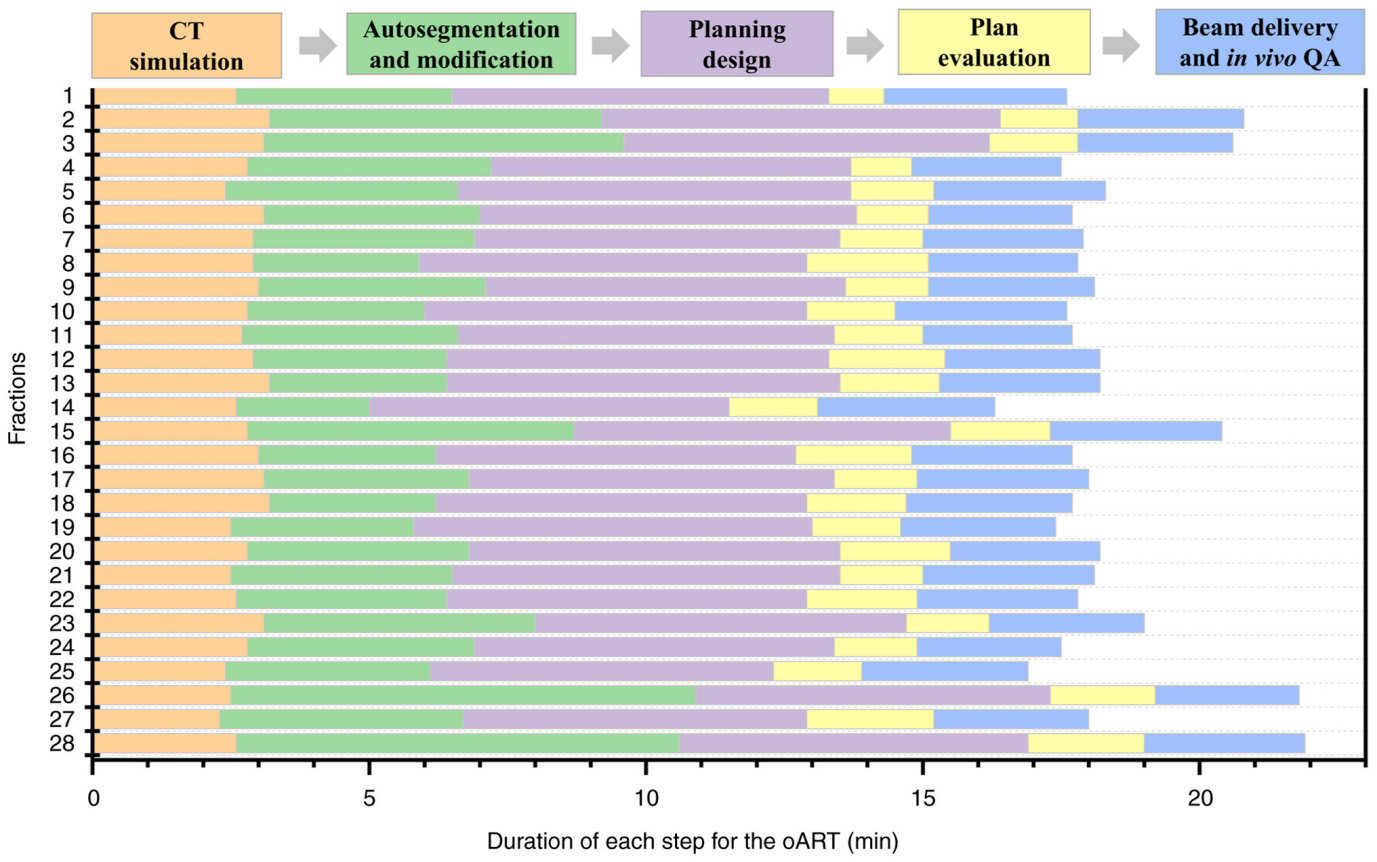
Time expended in each step of the oART workflow for the patient. oART, online adaptive radiotherapy; CT, computed tomography; QA, quality assurance.

**Figure 5.**
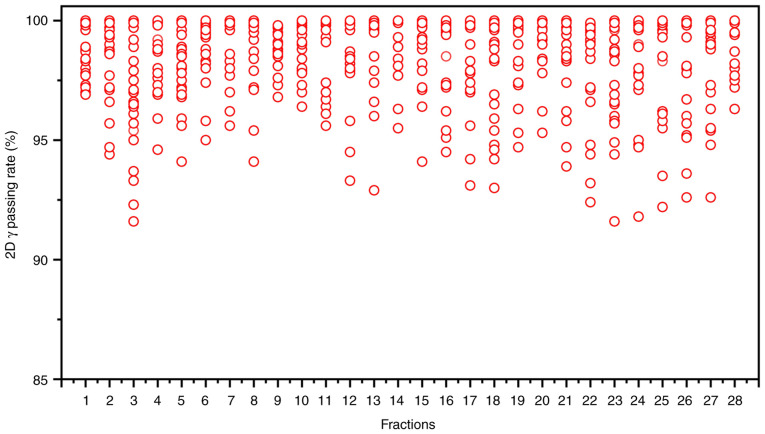
2D γ passing rates of *in vivo* QA for the oART fractions of the patient.

Achieving efficient and high-quality oART requires addressing several critical factors. First, high-quality imaging is critical for oART, as target contouring and dose planning depend on linear accelerator (linac)-acquired images. Conventional linacs use kilovoltage (kV) cone-beam CT (CBCT), but the low soft-tissue contrast (due to scattering artifacts) limits tumor visualization and forces reliance on bony landmarks for registration. CBCT also lacks sufficient resolution for precise target/OAR delineation and fails to provide accurate Hounsfield unit-based electron density data for dose calculation (11–14). The present study employed the uRT-linac 506c (United Imaging Healthcare Co., Ltd.), a CT-integrated linac with a 16-slice kV FBCT scanner. The FBCT delivers diagnostic-quality images comparable to planning CTs, enabling superior soft-tissue contrast for target/OAR delineation and reliable electron density mapping. By contrast, systems like Ethos enhance CBCT through iterative reconstruction with scatter correction, while Unity uses predefined electron density maps for synthetic CT generation. The second critical factor is an efficient and intelligent workflow. Anatomical shifts often occur within minutes or tens of minutes; thus, timely execution is essential for successful oART. The oART workflow relies on seamless integration of software and hardware, requiring multidisciplinary collaboration among radiotherapists, physicists and therapists. Key components include rapid contouring tools, efficient treatment planning systems and high-precision delivery equipment. Three oART platforms are currently implemented in clinical practice: The FBCT-guided uRT-Linac 506c (United Imaging Healthcare Co., Ltd.), CBCT-guided Ethos (Varian; Siemens Healthineers) and MR-guided Unity (Elekta Instrument AB). Empirical studies have validated the feasibility and time efficiency of these systems (15–17). Specifically, the uRT-Linac 506c and Ethos workflows require ~20 min for a complete oART cycle, whereas Unity necessitates >30 min. Efficiency in oART workflows is critically dependent on AI. Advances in AI have accelerated contouring and planning processes, primarily through automated segmentation of targets and OARs, and AI-driven treatment planning. To date, studies have demonstrated the utility of AI-based segmentation models and dose-prediction algorithms for automated planning (18–21). These tools reduce labor and time demands while enhancing the precision, efficiency and consistency of target delineation and dose distribution. In the present case, a dedicated AI segmentation model for cervical cancer based on multi-decoder and semi-supervised learning was implemented to enable automated target delineation during the patient's oART workflow (22). The dice similarity coefficient (DSC) for GTVp, CTV1 and CTV2 were determined to be 0.81±0.03, 0.90±0.02 and 0.92±0.01, respectively. Manual modification of the target during oART based on the auto-segmentation efficiently reduced time consumption, which was measured to be 4.31±1.43 min. For autosegmentation of OARs, a multiresolution VB-Net convolutional neural network was used, in which a cascade coarse-to-fine strategy was utilized to accelerate the contouring process (23), and all the contouring of OARs were clinically accepted without any modification. For the online adaptive plan, an adaptive optimization algorithm based on dose prediction was adopted. In the adaptive optimization algorithm, dose prediction and the original plan's clinical goal were utilized as inputs, allowing the algorithm to take into account patient-specific geometry, while clinical priorities contained in the clinical goal sheet provide explicit guidance. Notably, the first-pass rate for the adaptive plan was 100%. The whole process of oART for this patient was recorded as 18.38±1.40 min (Fig. 4). The third critical requirement for oART is robust online QA. In oART, the patient remains positioned on the treatment couch throughout the session, precluding conventional pre-treatment plan verification. Consequently, rapid and precise online QA systems are essential to validate treatment plans while minimizing overall workflow duration. In the present study, the electronic portal imaging device integrated into the linac facilitated *in vivo* QA. During beam delivery, the two-dimensional γ index was assessed at 30° intervals to verify beam accuracy, yielding a mean γ passing rate of 98.52±0.55% and a minimum γ passing rate of 91.60% (criteria: 3%/3 mm, 10% threshold; Fig. 5). Additionally, post-treatment three-dimensional dose reconstruction quantified the delivered dose, achieving a mean γ passing rate of 96.28±1.05% under the same criteria (Fig. 3D). These results confirm the precision and reliability of dose delivery during the oART process.

Notably, this patient showed an almost complete response on imaging with only the dose of external beam irradiation, and no cancer cells were even detected by postoperative pathology, as reported by the Department of Pathology (Fig. 1B). Throughout treatment and follow-up, the patient experienced no marked radiation-related toxicities, likely attributable to the precision of oART. While oART demonstrated efficacy in this case, its routine application across all treatment fractions remains impractical under current clinical workflows. Further research is warranted to establish standardized patient-specific criteria for initiating oART. Notably, histopathological confirmation in this case was only made possible due to treatment protocol modification (replacing BT with surgery) requested by the patient following EBRT, which is not routinely attainable under standard therapeutic guidelines. Consequently, the generalizability of these findings to broader patient populations remains uncertain. Further validation through additional clinical investigations is warranted, such as comparative assessments utilizing anatomical and functional imaging modalities to evaluate local tumor response between oART and non-oART cohorts post-EBRT, along with longitudinal monitoring of treatment-related toxicities and survival outcomes. Whether the BT dosage and frequency can be reduced or even entirely omitted following online adaptive EBRT in cervical cancer management remains a critical question that warrants rigorous clinical investigation. Comparative studies evaluating de-escalated BT protocols against standard-of-care regimens, with longitudinal assessment of local control rates and toxicity profiles, are imperative to validate this therapeutic paradigm shift.

In conclusion, the present case demonstrated the feasibility, safety and efficacy of FBCT-guided oART for cervical cancer. Notably, the workflow exhibited not only dosimetric superiority but also favorable tumor response and clinically tolerable toxicities. These findings advocate for the broader clinical adoption of oART in cervical cancer management to optimize local tumor control while mitigating radiation-induced toxicity to adjacent organs and systemic effects.

## Figures and Tables

**Table I.** Comparison of dosimetric parameters of PTV and organs at risk.

Parameter	Initial-Plan	IGRT-Plan	ART-Plan
PTV			
D_max_, cGy	5,375.14	5,815.58±106.01	5,356.02±21.25
D_98_, cGy	5,011.73	4,422.74±669.05	5,053.52±19.89
D_95_, cGy	5,047.39	4,825.80±191.45	5,087.15±15.04
D_50_, cGy	5,171.68	5,195.35±23.82	5,198.88±22.32
D_2_, cGy	5,279.86	5,334.00±16.63	5,304.06±26.12
CI^a^	0.92	0.79±0.05	0.92±0.01
HI^b^	0.05	0.18±0.13	0.05±0.00
V_100%_, %	95.94	88.99±4.40	98.51±0.82
Bladder-PTV			
V_40_, %	25.66	22.29±12.12	31.54±12.15
Rectum-PTV			
D_max_, cGy	4576.36	5076.71±248.57	4566.73±45.61
V_40_, %	16.94	33.03±19.05	16.96±3.39
Small bowel-PTV			
D_max_, cGy	4757.92	5368.87±208.65	4734.66±46.63
V_40_, %	4.68	5.06±2.00	4.46±1.06
Left femur head			
V_30_, %	5.88	6.81±1.01	6.00±0.81
V_50_, %	0.00	0.00±0.00	0.00±0.00
Right femur head			
V_30_, %	4.83	4.78±1.06	5.18±0.48
V_50_, %	0.00	0.00±0.00	0.00±0.00

a CI=(V_t,ref_/V_t_) × (V_t,ref_/V_ref_);

b HI=(D_2_-D_98_)/D_50_; CI, conformity index; V_t,ref_, target volume covered by the reference isodose line; V_t_, target volume; V_ref_, total volume covered by the reference isodose line; HI, homogeneity index; D_max_, maximum dose; D_98_, D_95_, D_50_ and D_2_, dose received by 98, 95, 50 and 2% of PTV; V_100%_, coverage of 100% prescription dose on PTV; V_30_, V_40_ and V_50_, volume ratios of 30, 40 and 50 Gy covering the corresponding organ at risk; PTV, planning target volume.

## Data Availability

The data generated in the present study may be requested from the corresponding author.
